# ChatGPT to enhance learning in dental education at a historically black medical college

**DOI:** 10.21203/rs.3.rs-3546693/v1

**Published:** 2023-11-07

**Authors:** Khandoker Rahad, Kianna Martin, Ihunna Amugo, Shania Ferguson, Angela Curtis, Anniya Davis, Pandu Gangula, Qingguo Wang

**Affiliations:** Meharry Medical College; Meharry Medical College; Meharry Medical College; Meharry Medical College; Meharry Medical College; Meharry Medical College; Meharry Medical College; Meharry Medical College

**Keywords:** ChatGPT, Dentistry, Dental Education, Medical School, Student Learning

## Abstract

In dental education of today, the didactic curriculum primarily relies on lecture-based courses, where instructors impart knowledge through presentations and discussions. The recent rise of powerful large language model-based tools, exemplified by ChatGPT (Chat Generative Pre-Trained Transformer), poses a great challenge to traditional dental education while simultaneously offering a unique resource and approach that potentially complements today’s teaching and learning, where existing widely available learning resources have often fallen short. In this paper, we used dental course materials, syllabi, and textbooks adopted currently in the School of Dentistry at Meharry Medical College to assess the potential utility and effectiveness of ChatGPT in dental education. We collected the responses of the chatbot to questions as well as students’ interactions with it for assessment. Our results showed that ChatGPT can assist in dental essay writing and generate relevant content for dental students, in addition to other benefits. The limitations of ChatGPT were also discussed in the paper.

## INTRODUCTION

The Chat Generative Pre-Trained Transformer (ChatGPT), along with other powerful large language model-based AI chatbots, is remarkably capable of generating text across a wide spectrum of formats: formal, informal, content, and creative writing, etc. They pose both challenges and opportunities for the world of education [1]. In the context of computer language courses, researchers have harnessed their potential to provide valuable support, including debugging assistance, bug prediction, and bug explanation, contributing to the resolution of programming challenges. Moreover, they offer suggestions and code corrections, drawing upon their comprehension of the intricate relationships between code and bugs [2]. Comparing with other educational resources, ChatGPT demonstrates clear advantages in terms of cost, speed, accuracy, customization, user-friendliness, seamless integration with existing tools, and scalability [3].

Firat *et al*. discussed the applications of ChatGPT within Learning Management Systems (LMS), which are platforms designed for delivering and managing online learning content and experiences [4]. ChatGPT enables students to quickly generate answers or essays with minimal research efforts, presenting a challenge for contemporary LMS [5]. Additionally, ChatGPT has been observed to generate fabricated facts and provide answers that are inaccurate or biased on certain topics. Another prominent concern is the chatbot’s limitations stemming from its training data, rendering it unaware of the latest events [6]. Instructors, however, may harness this limitation by requiring students to incorporate current events into their writing assignments.

Moreover, a recent study on the application of ChatGPT in the field of dentistry demonstrated its potential to facilitate precise and efficient diagnoses, ultimately alleviating the workload of dentists, who are increasingly relying on computer programs for decision making in contemporary dental practice [7]. Specifically, in the domain of two-dimensional radiographs in dentistry, where each pixel of a grayscale image represents the intensity or brightness of an object, ChatGPT, when coupled with other AI tools, is instrumental in elucidating patterns, segmenting teeth, and detecting caries, among other functions [7]. Furthermore, when compared to conventional treatment management methods, ChatGPT enables healthcare professionals to manage a higher patient load without compromising service quality. It allows dentists to monitor patients’ dental status more closely, offer detailed remote guidance for treatment, and modify instructions for subsequent steps, e.g., the repositioning of intra- and inter-maxillary elastics. In addition, it can aid in the early detection of issues, potentially weeks before the next scheduled appointment [9].

The rapid advancements in AI language models necessitate a shift in patient communication and the adaptation of dental education, including essay, thesis, and scientific paper writing. In this paper, we explore the potential of integrating ChatGPT into dental education to enhance dental students’ learning experiences. While ChatGPT has been evaluated in numerous educational contexts, its specific implications for dental education, a field known for its competitiveness and rigorous demands, remain insufficiently understood.

This study was conducted in the School of Dentistry (SOD) at Meharry Medical College (MMC). MMC, a historically black college/university (HBCU) and the first medical school for African Americans (AA) in the South, has trained over 40% of America’s AA dentists and produced 8% of the nation’s AA physicians. Notably, the majority of dental students and residents in MMC’s SOD are AA (constituting 80% of the student body) and three out of every four of them provide medical or dental services in urban or rural underserved communities. By leveraging ChatGPT to assist dental students in learning, this project can potentially enhance students’ academic performance, which, in turn, will empower MMC to better serve patients in underserved communities. Furthermore, this project was supported in part by a NIMHD supplement grant under award number U54MD007586 [10–12].

## MATERIALS AND METHODS

Dental terminologies, which were selected based on their prevalence in dental education, were used to evaluate ChatGPT. Incorrect terminologies, e.g., incorrect word order, improper capitalization and abbreviation, and the use of numerals in place of Roman numerals, were presented to ChatGPT to assess if it can recognize and rectify these dental-specific terms. Patient-related data was not used in any of our experiments. In addition, this study was conducted using version 3.5 of ChatGPT, which was the available version at the time and the one consistently utilized throughout this project.

Another category of data employed in our study comprises the test objectives of the National Board Examinations (INBDE), which was designed to aid dental boards in determining the qualifications of those seeking licensure to practice dentistry or dental hygiene. The ChatGPT generated outputs based on the INBDE codes for various dental subjects were evaluated by domain experts to verify their accuracy. In addition, ChatGPT was tasked with responding to a diverse range of queries, including general queries from dental students, assignments (including quizzes), dental literature-related questions, etc.

**Figure 1:**
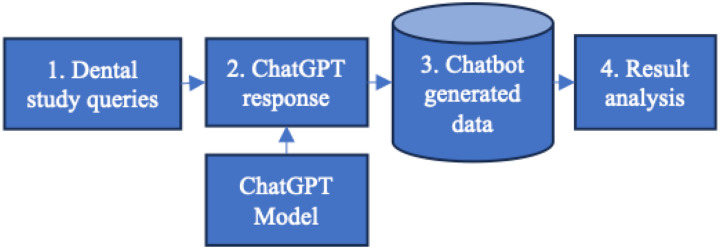
Visualization of data collection and analysis steps

Figure 1 provides an overview of our procedures for collecting and processing data generated by ChatGPT. These steps are elaborated below:

1. Dental study queries were created based on various categories of assessments in dental education.
2. Dental queries were executed using ChatGPT’s web interface.
3. ChatGPT generated responses were collected for downstream analysis.
4. The collected data was analyzed.

Six domain experts, who also serve as co-authors of this paper, were responsible for both generating questions for ChatGPT and verifying its responses. This group included five dental students and a professor from the School of Dentistry. For ChatGPT generated results, the experts employed a Likert scale to assess their accuracy quantitatively. On the data captured, descriptive analyses, including means, median, and frequencies, were performed.

To ensure reproducibility of the work, we validated some selected results. For some results, after conducting an initial experiment we asked similar set of questions a week later to check the robustness of ChatGPT’s responses. In addition, we followed the guideline as outlined in [13] in all our experiments, the results of which were presented in section below.

## RESULTS

### ChatGPT to Enhance Writing in Dental Education

Essays and reflective writings are an important assessment tool in dental curriculum. They are used widely as a means of developing students’ critical thinking skills and learning. With ChatGPT’s ability to produce creative writing being well documented in other fields [1], to assess the effectiveness of using ChatGPT to assist dental students in writing, here we focused on evaluating its capability of recognizing and correcting dental terminologies in student essays.

We collected essays from a group of dental students that describe dental histories of patients. Various types of errors that are commonly found in essays of dental students were added into these essays, such as incorrect alphabet order, improper capitalization and abbreviation, and numerals instead of Roman numerals. Table 1 shows some example errors, from which we can see that many capitalization errors, punctuation errors, spelling errors, subject-verb agreement errors, and other dental-specific errors require dental background to comprehend.

**Figure 2:**
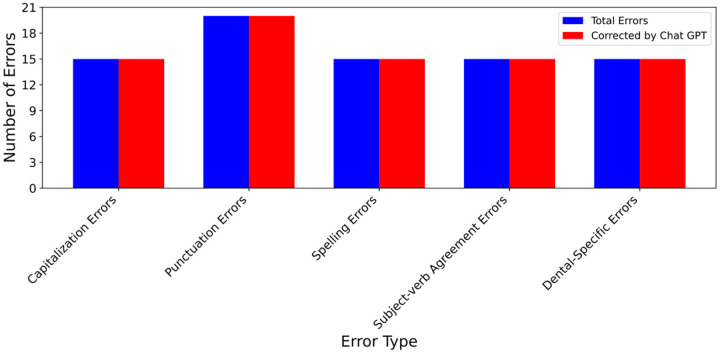
Various errors corrected by ChatGPT across essays on patients’ dental history

We applied ChatGPT to the essays that contains artificially added errors to check if it can identify and rectify these errors. Figure 2 provides the result of our assessment, in which blue bars present the total number of errors, red bars present the total number of errors corrected by ChatGPT, X-axis represents error types, and Y-axis number of errors. It shows in the Fig. 2 that ChatGPT successfully identified and corrected all the errors, indicating it can be a valuable tool for assisting dental students in writing.

### Accuracy of ChatGPT Responses

The ability of AI tools such as ChatGPT to consistently provide accurate responses to questions is vital in dental education and practice, because this can not only help identify dental issues in an effective manner, but also enhance the applications and adoptions of ChatGPT in dental education and practice to assist in dental assessment. On the other hand, inaccurate responses can create an image of untrustworthy or even be devastating, as precise and accurate results work as the key in dentistry.

To measure the accuracy of ChatGPT responses quantitatively, we formulated a rubric, which is presented in Table 2, and which associates each criterion for evaluating a ChatGPT response with a grade. For simplicity, the scale of the rubric ranges from 0 to 3, with 0 indicating that a statement is not reflected in a ChatGPT output at all, grade 1 indicating a statement-related information is present but with discrepancies, 2 indicating slightly changed or altered wording in a ChatGPT response, and scale 3 indicating a precise statement is found in a ChatGPT response (Table 2).

To make our experiment reproducible by other researchers, instead of protected clinical notes, we asked ChatGPT to answer questions for a selected set of published articles and then measured its accuracy by applying the rubric to its responses. Table 3 shows the main topics of a recently published paper [7] (1st column) and ChatGPT’s summary (2nd column) - ChatGPT was asked to provide key points of this paper. Evaluation of ChatGPT response is provided in the last two columns of Table 3, which shows a median accuracy 2.0 (or 2/3 = 66.67%), indicating ChatGPT’s ability to accurately extract and synthesize information from documents still needs to be improved.

Table 4 presents ChatGPT responses for three additional articles [7][8][9]. These articles cover various topics about ChatGPT: benefits, concerns, scientific discovery and innovation, etc. We asked ChatGPT questions for these articles and then graded its responses. The accuracy of ChatGPT responses in Table 4 shows that the highest rubric score is 2.0 (66.67%), the lowest one is 0.0, and the median score is 50.00%, indicating the overall performance of ChatGPT across these articles. From Tables 3 and 4, we can see that ChatGPT should be used with caution as the accuracy of its responses lacks consistency.

### Assistance in Test Preparation

With testing being a critical part of teaching and learning, next we evaluated if ChaGPT can assist dental students in testing preparation. In dental education, the INBDE Dental Board Exam is one of the most important steps on a student’s journey to becoming a dentist. Here, INBDE, which stands for National Board Examinations, was designed to assist dental boards in determining the qualifications of those seeking licensure to practice dentistry or dental hygiene.

For each INBDE code, we asked ChatGPT to generate a test question from a peer review resource. Its output was then evaluated by domain experts to confirm its relevance. Table 5 shows some example INBDE Test Objectives for dental examination and ChatGPT generated questions for them. As shown in Table 5, the subject areas in this assessment is broad and compressive, covering various aspects of dental education, including manual instructions for a class on amalgam cavity preparation in an operative dentistry class, analysis of objectives for a nutrition class, generation of study questions based on dental board question concepts from a periodontics class, interpretation of dental radiographs for a dental radiography class, and analysis of chapter summary topics from a pathology class textbook, etc.

The analysis of ChatGPT generated questions showed that it was capable of bringing diverse information together on a specific subject. In addition, some generated questions were inappropriate to be used directly for testing if without refinement, indicating that current version of ChatGPT still lacks higher-order intelligence that is required to correctly synthesize information. Despite its limitation, the questions and answers generated using ChatGPT still have some values. Some could potentially be used to help students identify subject areas that require additional efforts in their preparation for the INBDE test.

### Other Assessments

We also conducted other assessments of ChatGPT. For example, we asked ChatGPT about dental health benefits of dietary fibers. ChatGPT responded with some recommendations as key benefits of dietary fibers: they promote regularity, reduce risk of colon cancer, help manage blood sugar, and lower cholesterol level, etc. Additionally, for a question about a dentistry operation procedure about class 1 amalgam preparation, ChatGPT response is very close to a dental expert recommendation in the area: ChatGPT summarized that class 1 amalgam preparation is a routine restorative procedure in operative dentistry and the key to a successful restoration is careful cavity preparation and amalgam condensation, followed by careful carving and polishing of the restoration.

## DISCUSSION

In this paper, we explored the efficacy and prominent applications of ChatGPT in dental education. Our results demonstrated that ChatGPT can effectively recognize and correct dental terminologies and hence is a valuable tool for aiding dental students in writing. A potential limitation in our approach is that our assessment relied on manual evaluations, introducing an element of subjectivity, as different assessors may have varying interpretations of errors. This variability could impact the accuracy and consistency of the error analysis. Furthermore, this study focused specifically on five categories of errors. Other types of errors or linguistic aspects that could influence writing quality were not considered, which may constrain the comprehensiveness of our analysis.

Furthermore, similar as other language models, ChatGPT may inherit biases inherent in its training data. These biases can influence its comprehension and correction of errors, potentially resulting in inconsistencies or inaccuracies in the suggestions it offers. ChatGPT may also have inherent limitations in its grasp of complex grammar rules, contextual nuances, or domain-specific language. These limitations could impede its ability to accurately identify and rectify errors, particularly in complex cases or instances involving uncommon error types.

The scope of this study needs to be expanded to encompass a longitudinal assessment of ChatGPT’s sustained impact on student performance and learning outcomes. Furthermore, it is imperative to delve into the ethical considerations entailed in the adoption of AI tools, including ChatGPT, within educational contexts. Additionally, the exploration of the potential integration of ChatGPT into established dental education frameworks and platforms holds promise for enhancing practicality and accessibility for students. Another intriguing avenue for further investigation lies in comparing ChatGPT with conventional methods commonly employed in dental education, such as grammar and spelling correction tools.

Collecting feedback from a large cohort of dental students who have actively employed ChatGPT as an educational tool can yield invaluable insights into their individual experiences and perceptions. Additionally, gaining an understanding of the various ways students utilize ChatGPT, as well as their attitudes and overall satisfaction with the tool’s assistance, could inform meaningful enhancements for the future. By embarking on these areas of investigation, we can gain deeper insights into the potential benefits, limitations, and implications associated with the integration of ChatGPT in dental education.

## CONCLUSION

The recent rise of ChatGPT, a capable large language model-based AI tool, presents both opportunities and challenges in contemporary dental education. The educational framework in the School of Dentistry (SOD) at Meharry Medical College predominantly revolves around lecture-based courses, where instructors disseminate knowledge through presentations and discussions. To address the challenges posed by ChatGPT, this paper explored the potential utility and effectiveness of integrating ChatGPT into the dental education in the SOD.

In our evaluation of ChatGPT’s suitability for aiding dental students in writing, we observed its capability to effectively recognize and accurately rectify special dental terminologies, which require dental background for comprehension. This observation indicates ChatGPT can be a valuable writing assistant to help dental students with essay writing. Furthermore, we measured the aptitude of ChatGPT in responding to questions that we posed for a selection of published articles. Our assessment, based on a grading rubric, revealed a median accuracy of 50% ~ 67%. This suggests that while ChatGPT can extract and synthesize information from documents, there is room for improvement in its precision. We also investigated ChatGPT generated test questions to gauge their utility in assisting dental students in test preparation. We found some ChatGPT generated questions held potential value for students.

It is important to acknowledge that the manual assessments employed in our study have imposed a limit on the scale of this pilot study. Notwithstanding this limitation, we believe that this work provides important information that would be vital for future scaling-up studies.

## Figures and Tables

**Table 1:** Erroneous dental terminologies provided to ChatGPT for recognition and correction.

Error type	Examples incorrect terminologies provided to ChatGPT	Corresponding correct terminologies
Incorrect order	MDO, BO, DMO, BDO	MOD, OB, MOD, DOB
Capitalization & abbreviation	bw-xray	BWX
Capitalization	mod, fmx, Unc-15 probing instrument, Pa xray, pan xray	MOD, FMX, UNC, PA, PAN
Numerals instead of Roman numerals	Class 5 amalgam prep, Class 2 composite, Periodontics Stage 3, Mobility: Grade 1, Class 2 mobility	Class V, Class II, Stage III, Grade I, Class II

**Table 2:** Rubrics for evaluating ChatGPT responses to questions.

Grade	Scale Criteria	Description
3	Precise Statement	Verbatim statement without alteration or deviation.
2	Altered Wording, Same Meaning	Different wording but retains the original intent and meaning.
1	Related but with Discrepancies	Vague, adds extra info, partially relevant, or omits some original information.
0	Not Presented in the ChatGPT	Statement did not exist at all.

**Table 3:** Assessing the accuracy of ChatGPT’s summary of a published article [7].

Main topics in the article	ChatGPT Response	Rubric Value	Accuracy %
LLMs for patient info & diagnosis	Helps describe symptoms & history	2	66.67%
LLMs for scalable telemedicine	Convenient & cost-effective access	2	66.67%
LLMs for real-time translation	Information not found	0	0%
ChatGPT not for medical guidance	Not the most suitable tool	2	66.67%
ChatGPT for insurance preauthorization	Verifies insurance info	2	66.67%
LLMs in dental schools	Personalizes learning 24/7	1	33.33%
**Median**		2	**66.67%**

LLM: Large Language Model

**Table 4:** Analysis of ChatGPT response accuracy based on articles [7][8][9].

Statements in published articles	ChatGPT Responses	Accuracy
**Article 1** Benefits of ChatGPT		
Personalized learning and adapt to individual student needs	Personalized experiences for students	66.67%
Freeing up educators to focus on important issues	Improved efficiency and productivity for educators	33.33%
**Article 2** Accelerated Scientific Discovery and Innovation		
Access to wide range of educational resources	Enhanced accessibility to education	66.67%
**Article 3** Concerns on ChatGPT Response		
AI biases and discrimination	Bias potential in Artificial Intelligence algorithms	66.67%
Decrease in human interaction and critical thinking	Overreliance on AI reduces critical thinking	66.67%
Plagiarism	Data privacy and ownership concerns	33.33%
AI’s effectiveness in generating quality research papers	-	0.00%
Ethical concerns: transparency, accountability	Lack of transparency and accountability	33.33%
**Median**		**50.00%**

**Table 5:** INBDE test questions generated by ChatGPT.

INBDE Test Objective	Peer-Review Resource	Example Question
CC#04 - Use clinical and epidemiological data to diagnose and establish a prognosis for dental abnormalities and pathology	Journal of Oral Pathology and Medicine	How can oral pathologists use molecular methods to improve the diagnosis and prognosis of oral cancer?
CC#05 - Recognize the normal range of clinical findings and distinguish significant deviations that require monitoring, treatment, or management	Journal of Dental Research	How do gingival crevicular fluid biomarkers differ in healthy patients compared to those with periodontal disease?
CC#06 - Predict the most likely diagnostic result given available patient information	Journal of Endon tonics	Can radiographic findings predict the success of non-surgical endodontic treatment?
CC#07 - Interpret diagnostic results to inform understanding of the patient’s condition	Journal of Prosthetics Dentistry	How can cone bear computed tomography be used to interpret implant placement and its effects on adjacent teeth?
CC#10 - Select the diagnostic tools most likely to establish or confirm the diagnosis	Journal of Periodontology	What is the most effective diagnostic tool for identifying periodontitis in its early stages?
CC#12 - Formulate a comprehensive diagnosis and treatment plan for patient management	Journal of Oral and Maxillofacial Surgery	What is the recommended treatment plan for a patient with a mandibular fracture?
CC#13 - Discuss etiologies, treatment alternatives, and prognoses with patients so they are educated and can make informed decisions concerning the management of their care	Journal of American Dental Association	What are the potential complications of a root canal procedure, and how can they be addressed?
CC#22 - Prevent, diagnose, and manage periodontal diseases	Journal of Clinical Periodontology	What are the most effective non-surgical periodontal treatments for managing periodontitis?
CC#41 - Evaluate scientific literature and integrate new knowledge and best research outcomes with patient values and other sources of information to make decisions about treatment	Journal of Evidence-Based Dental Practice	How can clinicians use evidence-based dentistry to make treatment decisions for patients with temporomandibular joint disorders?
